# Gastric and Duodenal Ulcers

**Published:** 1890-03

**Authors:** Clarence King

**Affiliations:** Machias, N.Y.; Physician to the Cattaraugus County Alms House and Insane Asylum


Buffalo Medical and Surgical Journal

VOL. XXIX. MARCH, 1890. NO. 8.

### Original Communications.

### GASTRIC AND DUODENAL ULCERS.

By CLARENCE KING, M. D., Machias, N. Y.

Physician to the Cattaraugus County Alms House and Insane Asylum.

A POSITIVE diagnosis of gastric or duodenal ulcer is conceded by all authors to be difficult in the great majority of cases. This is due, first, to the hidden position of the organs involved, thus making a satisfactory examination difficult; second, to the frequent resemblance which the various functional and other structural diseases, notably cancer, show to ulcer; and third, to the great variation which occurs, even in the most constant and characteristic symptoms. Indeed, leaving out of consideration the evidence afforded by a chemical examination of the secretions of the stomach, we may say that there is no symptom of ulcer universally present in this disease, and no one but may be developed when ulcer does not exist. There is, however, evidence to show that ulceration in this position does occur with greater frequency than has been generally believed. Many cases doubtless recover without the true condition ever being suspected, while many fatal cases are credited to other causes and the diagnosis never corrected by autopsy. Welch has collected the histories of 32,052 autopsies in which the stomach was examined, and in these there were 1522 open ulcers or cicatrices present, a percentage of nearly 5. But while gastric ulcer is thus shown to be a relatively common disease, true duodenal ulcer is very rare. Pepper has stated,[1] as recently as May last, that it is doubtful if more than 70 authenticated cases are on record, to which number, however, he adds another verified by autopsy, and one believed to be duodenal, but in which recovery took place.

---

1. Journal of the American Medical Association, May 25, 1889. See also *Medical and Surgical Reporter*, February 15, 1890, where he reports another probable case.

The etiology of gastric and duodenal ulcer is clouded in obscurity. Many theories have been advanced to explain their causation, some of which appear tenable while others have long since been rejected. The best known of these theories, perhaps, is that of Virchow, who sought to explain the causation of ulcers in these situations by the occurrence of minute emboli or thromboses, and consequent stasis in the blood current in circumscribed areas with subsequent digestion of these areas by the gastric juice. Although apparently supported by abundance of pathological and experimental data, there are serious anatomical objections to the acceptance of these views. The anastomoses of the gastric and duodenal vessels are very abundant, and it is difficult to conceive how an embolus or a thrombus could seriously interfere with the circulation. Besides, why should the obstruction occur so much more frequently in the stomach and first horizontal third of the duodenum than elsewhere?

Extensive burns of the abdominal walls are well-recognized causes of duodenal ulcers, and a few cases are on record in which gastric ulcers have been produced in this way. In such cases the ulcer is produced traumatically and the duodenum, from its more exposed position, suffers the oftenest. It is difficult to give a satisfactory explanation why the colon, which is still more exposed to external traumatic influences, should so universally escape in these injuries without ulcer. Blows and kicks in the region of the stomach, the ingestion of hot foods and drinks, and the introduction of foreign substances into the circulation have each been experimentally shown to be capable of producing ulcer of the stomach. Various other causes have been assigned, some of which are supported clinically and are, undoubtedly, occasional causes; but others may be regarded only as proofs of the imaginative genius of the human mind.

The following cases are here reported as being of clinical interest:

CASE I.—GASTRIC ULCER. PAIN, VOMITING, HEMATEMESIS. DEATH FROM EXHAUSTION AFTER NINE MONTHS.

Mr. A. W., age 54, a carpenter by trade, always been very strong and healthy. Mother died of cancer. No tubercular or syphilitic taint.

About the first of June, 1886, he began to suffer with symptoms of indigestion. His food distressed him, and at times he suffered some pain in the stomach, which came on a short time after eating, and continued for two or three hours. In the interval he would be entirely free from pain. His bowels were constipated, tongue furred, appetite poor, nausea and occasionally vomiting present.

These symptoms continued to grow worse until he was obliged to seek treatment. Often, while at his work, the pain would come on and be so severe as to oblige him to lie down for a period varying from twenty minutes to an hour. After this he would be able to resume his work. He gradually lost flesh and became anemic. He was put on an anti-dyspeptic treatment which, he thought, afforded some relief.

About December 1st he vomited nearly three pints of bright red blood and two days afterward about as much more. As a consequence his countenance became very much blanched. He was weak and very nervous with a small and rapid pulse. Upon deep pressure a small, indistinct tumor could be felt and, as his countenance had assumed a somewhat cachectic appearance, he was thought to have a cancer of the stomach. No chemical examination was made of the secretions from the stomach. He was kept on his back and ice applied to the epigastrium. Hypodermic injections of ergotin and morphia were given, and nourishment administered by the rectum. After this emesis seldom occurred, but occasionally a small amount of blood was found in the vomited matter, and he still experienced considerable pain. He began slowly to improve and, after three weeks, he was allowed a tablespoonful of milk, *per orem*, every two hours. This caused him no distress and the amount was gradually increased until, finally, he was allowed small quantities of solid food.

About February 1st, he began to fail again and required more morphia to relieve the pain. He vomited occasionally, but no blood. We could not see as he was losing flesh, but his countenance was pinched and anemic and decidedly cachectic. He died on the 15th, six weeks after his last hemorrhage.

The autopsy revealed an ulcer, as large as a quarter of a dollar, on the greater curvature of the stomach, near the pylorus, with induration to the extent of an inch around it. The liver was much enlarged; other organs normal.

This case presents nothing novel in its clinical history and may be taken as typical of the disease. With it we will compare other cases which have come to our observation.

CASE II.—GASTRIC ULCER OF MANY YEARS' DURATION. REPEATED ATTACKS OF ACUTE PAIN AND VOMITING; HEMORRHAGE, PERFORATION, DEATH.

Mr. W. C., age 66, a farmer and hard-working man. Drank some when young, but later became strictly temperate.

When a child eight or ten years of age, he began to have trouble with his stomach which occasionally amounted to positive pain.

This gastric uneasiness or pain occurred for the most part during the intervals of digestion, and he was in the habit of carrying a crust of bread in his pocket to eat when his stomach felt bad, and for some years this would relieve the pain; later he carried a can of sweet milk or buttermilk for the same purpose. When still a young man he began to have acute attacks of severe gastric pain, accompanied by emesis, which the ingestion of food increased rather than alleviated. These attacks occurred without apparent cause, lasted from three to five days, and while they continued he was unable to take any food into his stomach on account of the pain which it occasioned, requiring large doses of morphia to allay it. He was unable to rest in bed for more than a few minutes at a time, and the little sleep he obtained was mostly secured when sitting upright in a chair with his body bent forward and his head resting on a table or chair in front of him. All the nourishment he took during the attacks was by the rectum. He would declare at each attack that he was going to rely entirely upon rectal alimentation even for six months to obtain a permanent cure for his disease, but each time would return to nature's method of taking food soon after getting better. He vomited frequently, sometimes blood more or less changed by the gastric secretions, and always felt easier after vomiting. Often, when in the greatest pain, he would put his finger far down into his throat for the purpose of provoking emesis. When free from pain he dreaded to move for fear of bringing it on again.

His last attack occurred the first of November, 1886. It resembled in all particulars those which he had suffered before, except that it followed more closely upon the one that preceded it. Hypodermic injections of morphia in one-quarter grain doses were required every two hours to relieve, to any extent, the pain. He vomited repeatedly, bringing up each time the dark, coffee-ground matter, indicative of hemorrhage. He rapidly lost strength and became very anemic. His pulse became very weak and irregular. Ergotin and digitalin were given him hypodermically and whiskey by the rectum. After suffering intense pain for about three days he gradually became easier and was able to lie down in bed, the moderate pain which he still had being transferred to his abdomen. His voice was weak; his features pinched; his extremities cold. His mind was clear and he conversed freely with his family. He continued in this condition for nearly two days, vomiting occasionally, and, finally, died in syncope on the 8th.

*Autopsy*. Upon opening the abdominal cavity, the stomach and intestines were found firmly bound together and to the other organs in this region, the result of inflammation. Adhesions existed between the stomach and the pancreas, diaphragm, meso-colon, liver and intestines. An ulcer, the shape of a three-leaf clover, each third being an inch and a half in diameter, was found on the greater curvature of the stomach, near to but not involving the pylorus. At the bottom of the part which was situated on the anterior wall was a perforation about an eighth of an inch in diameter, through which fluids had passed into the abdominal cavity. In trying to separate the posterior wall from the pancreas an opening was made into the stomach, and, upon closer examination, it was discovered that the wall of the stomach was entirely wanting at this point and that the ulcer extended quite deeply into the substance of the pancreas. The stomach was smaller in size than normal and the walls thickened and inflamed, especially for some distance around the ulcer and in the pyloric region. The pyloric outlet was opened sufficiently to admit the index finger and the organ was empty, except the mucus, which bathed the surface of the ulcer and contiguous walls. No cicatrix was found in any part.

This case is of interest from the size and shape of the ulcer and the duration of the disease. My father, the late Dr. Thomas J. King, made a diagnosis of ulcer nearly thirty years ago and I am assured that his condition existed practically the same for some years before that time. His earliest symptoms point to functional disease, and it is impossible to state just when that gave place to structural changes.

Some physicians might regard this as an example of the recurrent form of ulcer in which one ulcer succeeds another. In such cases numerous cicatrices are found indicating the site of former ulcers. But as no cicatrices were found I consider it as a single ulcer in which the destructive alternated with the reparative process, and in which ulceration attacked the newly formed cicatricial tissue before it became firm or the process of cicatrization was completed.

### CASE III.—PROBABLE GASTRIC ULCER IN A CHILD SIX YEARS OLD, THE RESULT OF PRESSURE. RECOVERY AFTER REMOVAL OF THE PRESSURE.

This patient was under my care at the Cattaraugus County Alms House.

Some months before being admitted to this institution he fell and injured his spine so as to make it necessary for him to wear a plaster-of-Paris jacket fitted with a jury mast. He improved under this treatment and was soon able to run around the room, inconvenienced somewhat, of course, by his confinement. He was very active and, owing to the small size of his hips, it was difficult to make a jacket that would keep its place and not work downward. After being under my care for a few weeks he began to have attacks of vomiting and said it hurt him in his stomach, especially after taking food even milk causing him distress. For this reason the attendants found it difficult to coax him to take any nourishment whatever. He rapidly lost strength and weight. Occasionally he vomited small amounts of blood, but it was never present in large quantity. He had no fever. I removed the jacket and applied a new one, making it tight around the hips but leaving it loose over the stomach. His food was limited to milk and barley water, which he took in small quantities by the stomach. He began to improve and, in three weeks, was free from pain, had ceased vomiting, was eating well and had regained some of his lost flesh. As the vertebral injury had likewise improved and as he was able to carry his head without artificial support the jacket was removed, and he was allowed to return to his parents in a distant town.

This case lacks the positive evidence of an autopsy to confirm the diagnosis of ulcer, but I have thought that an ulcer would explain the symptoms more satisfactorily than any other malady. If we accept this diagnosis the case is doubly interesting: First, from the age of the patient, and second, from the probable cause of the ulcer, viz., pressure from an improperly applied jacket.

The treatment of the case, aside from that already indicated, was simple. He was given opiates to control the pain, cod liver oil, and wine of pepsin with dilute muriatic acid in medium doses. It would have been well-nigh impossible to have succeeded at rectal alimentation and the attempt was not made. I have never heard from the patient since he left the institution.

CASE IV.—DUODENAL ULCER.—PAIN; VOMITING; PERFORATION; DEATH.

This case occurred in the practice of my father, the late Dr. Thomas J. King, and was seen professionally by me but once before his death.

Mr. H. P., age 68, had always been a hard-working man, and, in his younger life, was very strong and healthy. Later he became a vegetarian in his diet, and lived for years almost entirely upon grahame, suffering more or less frequently with attacks of pain at the pit of the stomach, which were always attributed to some foolish venture upon forbidden food. These paroxysms sometimes lasted several days and were generally accompanied by vomiting, but the character of the matter thus expelled is not made clear by the history which I am able to obtain.

In October, 1887, he had one of these attacks and was obliged to call a physician. He suffered intense pain in the epigastric region and vomited repeatedly, the matter expelled presenting the characteristic coffee-ground appearance. He was unable to retain any food in his stomach and, for some days, was nourished by rectal enemata. He also passed this same dark, coffee-ground fluid by the rectum, but this occurred only once or twice and was not very great in amount. He became pale and restless and refused stimulants. His pulse was small and rapid, his tongue furred, his extremities cold. The bowels were distended and tympanitic and exquisitely tender to pressure; the pain radiated through the whole abdomen, to the back between the shoulders, and to the sacral region. He died December 11th.

Autopsy made by myself: Patient well nourished, the subcutaneous fat being preserved. Stomach partially distended with fluid of the same character as that vomited. Pyloric end of stomach and duodenum bound to neighboring structures by adhesions. An ulcer, the size of a nickel, was found on the anterior wall of the duodenum, an inch below the pylorus, and at the bottom of the ulcer a perforation through the remaining coats of the bowel. Stomach somewhat inflamed at the pyloric extremity, pylorus patulous; other organs normal.

